# Editorial: Integrative single-cell and spatial multiomics: charting cellular architecture and disease microenvironments

**DOI:** 10.3389/fbinf.2026.1983096

**Published:** 2026-09-08

**Authors:** Wenchong Chen, Jingjing Lu, Wancheng Guo, Boyu Li, Hang Zhang, Yilin Wang, Zhaokai Zhou, Ran Xu

**Affiliations:** 1 Department of Urology, The Second Xiangya Hospital of Central South University, Changsha, Hunan, China; 2 Department of Neurology, The First Affiliated Hospital of Zhengzhou University, Zhengzhou, Henan, China; 3 The Xiangya School of Medicine, Central South University, Changsha, China; 4 National Clinical Research Center for Metabolic Diseases, The Second Xiangya Hospital of Central South University, Changsha, Hunan, China

**Keywords:** data integration, disease microenvironment, multiomics, single-cell, spatial transcriptomics

## Introduction

Cellular heterogeneity and spatial organization are crucial to tissue function, and their disruption or remodeling is associated with disease onset and progression (Gulati et al., 2025). Integrative single-cell and spatial multiomics technologies offer a new lens through which to view this complexity. Single-cell transcriptomics can identify cell populations and transcriptional states that are obscured by bulk RNA sequencing (bulk RNA-seq), whereas spatial transcriptomics preserves the spatial context of cells and gene expression. When combined with complementary omic data, these approaches enable analyses of cell states, cell-cell interactions, and microenvironmental niches across tissue space (Longo et al., 2021; Jain and Eadon, 2024). They provide a framework for examining how cellular organization contributes to development, physiology, and disease.

This Research Topic includes six Original Research articles that examine cancer, neurological disorders, and inflammatory and metabolic diseases using single-cell, spatial transcriptomic, and integrative multiomic approaches. Collectively, these studies illustrate recent advances and applications of single-cell and spatial multiomics in disease research.

## Single-cell analysis of cell-state transitions and immune remodeling in breast cancer

Verrina et al. used a newly generated single-cell RNA sequencing (scRNA-seq) dataset of invasive ductal breast carcinoma, pseudotime analysis, and interpretable decision-tree models to investigate an epithelial-to-mesenchymal transition (EMT)-related continuum of cell states. They identified genes and expression thresholds associated with these transitions. Tumor progression involves dynamic transitions in cancer-cell states and continual remodeling of the surrounding immune microenvironment. Khan S. et al. integrated breast cancer scRNA-seq data from normal breast tissue, primary tumors, tumor-draining lymph nodes, and peripheral blood to examine immune remodeling. Tumors were enriched in effector CD8^+^ T cells, whereas tumor-draining lymph nodes contained higher proportions of naïve CD4^+^ T cells, naïve B cells, and memory B cells. Based on cell–cell communication analysis, TCGA-BRCA survival analysis, and peptide design, the authors proposed that BTLA–HVEM and CXCL12–CXCR4 may contribute to resistance to PD-1/PD-L1 blockade. These axes may also represent candidate biomarkers and therapeutic targets.

## Spatial transcriptomic analysis of brain injury and demyelination

Integrating spatial transcriptomics with single-cell data enables analysis of disease-associated cellular changes across distinct anatomical regions (Liu et al., 2024). This strategy was applied to models of intracerebral hemorrhage and demyelination. Zhang et al. integrated bulk RNA-seq data from human perihematomal tissue, scRNA-seq data from a mouse intracerebral hemorrhage (ICH) model, and time-series spatial transcriptomic data. They identified *HK2*, *HSPA5*, and *TNF* as candidate regulators linking glucose metabolism and neuroinflammation. Spatial analysis showed time-dependent *HK2* expression in microglia and a synchronized decline of *HK2*, *HSPA5*, and *TNF* in perihematomal regions at day 7 post-ICH. Tsai et al. integrated bulk RNA-seq, snRNA-seq, and spatial transcriptomics to characterize demyelination and remyelination in the cuprizone model. The study found that oligodendroglia and microglia exhibited the most prominent transcriptional changes, and cuprizone-associated subclusters of both cell types localized to the corpus callosum, a classical lesion site. During recovery, mature oligodendrocytes nearly returned to a control-like state, whereas microglia retained a demyelination-associated phenotype. Spatial analysis also identified cuprizone-associated remodeling of inferred glial cell–cell communication.

## Comparative analysis of spatial transcriptomic annotation methods in non-small cell lung cancer (NSCLC)

Sequencing-based spatial transcriptomics generates a spot-by-gene matrix structure that generally lacks single-cell resolution (Gaspard-Boulinc et al., 2025). Given this limitation, the cell-type composition of individual spots must be inferred from mixed transcriptional signals, and differences among deconvolution methods may affect subsequent interpretations of tissue spatial organization (Li et al., 2023). Wu et al. compared four commonly used cell-type annotation methods—Seurat, CARD, RCTD, and SPOTlight—across 45 NSCLC spatial transcriptomic samples comprising 328,561 spots. Estimated cell-type composition differed among the methods, with CARD and RCTD showing the greatest concordance. Despite these differences, cross-method consensus analysis identified increased metabolic and translational activity in tumor-proximal regions, and independent scRNA-seq data supported these transcriptional signatures. The study indicates that combining multiple annotation methods can enhance the identification of the spatial organization and associated biological features of complex tumor tissues.

## Integrated multiomic analysis and experimental validation in gout and metabolic syndrome

Single-cell and multiomics analyses can provide mechanistic insights into disease development and progression, but their clinical translation remains distant, and experimental validation is needed to ensure the biological relevance of these findings (Skinnider et al., 2025). Shao et al. integrated bulk transcriptomic and scRNA-seq data and identified 261 differentially expressed genes, including 19 hub genes shared by gout and metabolic syndrome. Single-cell analysis showed that the hub gene *JAK1* was predominantly expressed in NK cells, whereas *CSF1R* was predominantly expressed by monocytes in gout and by macrophages in metabolic syndrome; the VISFATIN signaling pathway was highly active in both diseases. *In vitro* experiments further confirmed elevated expression of *JAK1*, *CSF1R*, and *NAMPT*, together with activation of the JAK–STAT pathway, in cell models of both diseases. These findings provided experimental support for the expression patterns and candidate pathways identified through multiomics analyses, thereby supporting the use of multiomics approaches to identify potential intervention targets.

## Conclusion

This Research Topic introduces six Original Research articles in the field of integrative single-cell and spatial multiomics approaches, and demonstrates how these multiomics technologies can be used to investigate cellular heterogeneity and tissue architecture in various diseases. In the near future, as single-cell sequencing methods become more widespread, the resolution of spatial transcriptomics improves, and methods for analyzing sequencing data continue to advance, these technologies will enable us to explore disease progression and pathogenesis with greater accuracy, supporting the eventual application of these approaches in clinical research (Wang et al., 2026).

## References

[B1] Gaspard-Boulinc L. C. Gortana L. Walter T. Barillot E. Cavalli F. M. G. (2025). Cell-type deconvolution methods for spatial transcriptomics. Nat. Rev. Genet. 26, 828–846. 10.1038/s41576-025-00845-y 40369312

[B2] Gulati G. S. D’Silva J. P. Liu Y. Wang L. Newman A. M. (2025). Profiling cell identity and tissue architecture with single-cell and spatial transcriptomics. Nat. Rev. Mol. Cell Biol. 26, 11–31. 10.1038/s41580-024-00768-2 39169166

[B3] Jain S. Eadon M. T. (2024). Spatial transcriptomics in health and disease. Nat. Rev. Nephrol. 20, 659–671. 10.1038/s41581-024-00841-1 38719971 PMC11392631

[B4] Li H. Zhou J. Li Z. Chen S. Liao X. Zhang B. (2023). A comprehensive benchmarking with practical guidelines for cellular deconvolution of spatial transcriptomics. Nat. Commun. 14, 1548. 10.1038/s41467-023-37168-7 36941264 PMC10027878

[B5] Liu M. Ji Z. Jain V. Smith V. L. Hocke E. Patel A. P. (2024). Spatial transcriptomics reveals segregation of tumor cell states in glioblastoma and marked immunosuppression within the perinecrotic niche. Acta Neuropathol. Commun. 12, 64. 10.1186/s40478-024-01769-0 38650010 PMC11036705

[B6] Longo S. K. Guo M. G. Ji A. L. Khavari P. A. (2021). Integrating single-cell and spatial transcriptomics to elucidate intercellular tissue dynamics. Nat. Rev. Genet. 22, 627–644. 10.1038/s41576-021-00370-8 34145435 PMC9888017

[B7] Skinnider M. A. Courtine G. Bloch J. Squair J. W. (2025). A clinical road map for single-cell omics. Cell 188, 3633–3647. 10.1016/j.cell.2025.06.009 40645168

[B8] Wang Z. Guo F. Sun H. Kong Y. Wang Z. Tao J. (2026). Clinical translation of spatial omics. Nat. Rev. Bioeng. 4, 699–701. 10.1038/s44222-026-00458-y

